# Uncovering Sexual Differences in the External Morphology, Appendicular Muscles, and Internal Organs of a Fossorial Narrow-Mouth Frog (*Kaloula borealis*)

**DOI:** 10.3390/ani15142118

**Published:** 2025-07-17

**Authors:** Xiuping Wang, Meihua Zhang, Wenyi Zhang, Jianping Jiang, Bingjun Dong

**Affiliations:** 1 College of Life Sciences, Shenyang Normal University, Shenyang 110034, China; wxiuping0523@163.com (X.W.); zhangwy0523@163.com (W.Z.); 2 Chengdu Institute of Biology, Chinese Academy of Sciences, Chengdu 610213, China; jiangjp@cib.ac.cn

**Keywords:** sexual dimorphism, functional morphology, 3D reconstruction, tissue mass, adaptations

## Abstract

Sexual dimorphism is prevalent among animals, influencing both functional morphological traits and behavioral performances. In this study, we investigated the sexual differences in the morphological parameters of *Kaloula borealis* (Anura, Microhylidae) during the breeding season using 48 specimens. Our results reveal that among the 16 external morphological traits, females had significantly larger snout-vent length and eye diameter than males. The former presumably contributes to enhancing fertility, while the latter is associated with larger body size. Males exhibited significantly greater head width and thigh width than females, which may be related to accessing a wider range of food sources and enhancing their locomotor ability, respectively. Among the 32 appendicular muscles, 10 displayed significant sexual dimorphism in dry mass, suggesting divergent reproductive strategies between the sexes. Among the eight internal organs analyzed, males possessed significantly heavier hearts and lungs than females, which is likely an adaptation to higher metabolic demands and calling behavior. Collectively, our findings demonstrate that sex-specific differences in external morphology, muscle mass, and internal organ mass reflect distinct ecological and reproductive adaptations between males and females and contribute to the phenotypic diversities in Anura.

## Simple Summary

We investigated the sexual differences in the external morphological characteristics, appendicular muscle mass, and internal organ mass of *Kaloula borealis* during the breeding season. The snout-vent length and eye diameter were significantly larger in females than in males. Males exhibited significantly greater head width and thigh width than females. The dry mass of ten appendicular muscles showed significant differences between the sexes. In addition, males had significantly heavier hearts and lungs compared to females. This study presents the detail and comprehensive analyses of sexual dimorphism in a fossorial anuran species, enhancing our better understanding of the anuran reproductive strategies.

## 1. Introduction

Sexual dimorphism, the phenotypic differences between males and females of the same species, is prevalent in animals [1]. Selection acting on sexually dimorphic traits can affect both functional morphological traits and behavioral performances, playing a crucial role in understanding patterns and processes at micro- and macroevolutionary scales [2,3,4]. Several hypotheses have been proposed to explain the ecological and evolutionary origins of sexual dimorphism. Empirical studies and life history theory classify these into three non-mutually exclusive selective mechanisms, namely, the sexual selection hypothesis, the fecundity selection hypothesis, and the niche divergence hypothesis [5]. Notably, the interplay between competition and mate choice in sexual selection drives the rapid and pronounced evolution of sexually dimorphic traits [6]. Sexual selection theory suggests that sexual phenotypic differences promote the evolution of morphology and behavior to enhance reproductive success, including resource control, intrasexual competition, and social dominance [7,8,9]. Fecundity selection emphasizes sex-specific energy allocation toward reproduction [10]. The niche divergence hypothesis proposes that dimorphism may evolve to reduce intersexual competition for resources [11].

Morphometry is a useful tool to determine species diversity by measuring the morphological traits, and to provide insights into reproductive strategy, fitness optimization, and ecological adaptation [12,13]. A prominent example is sexual size dimorphism in anurans, with adult females being significantly larger than males in most studied species [14,15,16]. The larger body size of females can be explained by fecundity selection. This is because larger body size correlates with increased abdominal cavity volume, enabling more or larger eggs, and consequently enhancing reproductive output [17]. Studies have shown a significant positive relationship between functional muscle mass and reproductive success in both sexes [18]. When adjusted for body size, males typically exhibit more developed appendicular muscles. Functionally, the heavier forelimb muscles provide greater strength to grip females more tightly and to prevent being displaced by other males, thereby improving mating success [19]. Beyond fundamental locomotion, like swimming and jumping, hindlimb muscles play a critical role in competitive behaviors, such as kicking and defensive maneuvers, with well-developed musculature conferring reproductive advantages. To date, research on sexual dimorphism in external morphology and appendicular musculature has been focused on semi-aquatic [20,21,22,23], terrestrial [24,25,26], and arboreal anuran species [27].

Selective pressures can also drive sexual dimorphism in internal organs [28]. Organ size variation is closely related to the storage and metabolism of energy [29], making the study of sex-specific differences in organ mass crucial for understanding functional dimorphism, and evolutionary adaptations in internal anatomy [30]. Current quantitative research on internal organ dimorphism mostly concentrates on function changes across geographical locations, environmental gradients or seasonal variations [29], development and differentiation [31], and morphology and histology [32,33]. However, sex-specific variations in organ mass during the reproductive period remain poorly studied.

*Kaloula borealis* (Barbour, 1908) [34] (Anura: Microhylidae) is a fossorial species that uses its hindlimbs for digging. It is distributed in northern China and the temperate region of Korea, with its type locality in Dandong City, Liaoning Province, China [34]. This species is an explosive breeder with axillary amplexus, and its reproductive period occurs from July to August. Currently, studies have been conducted on different regions of its distribution range. There are differences in the growth rate between males and females [35]. In addition, research on *K. borealis* has focused on comprehensive osteological examinations [36], distribution and biological characteristics [37], genetic analysis [38], age structure [39], and ecology and habitat restoration [40]. We speculate that this explosive breeder exhibits significant sexual dimorphism, with males possessing more developed appendages and internal organs to adapt to amplexus, calls, and other reproductive behaviors. In this study, we will comprehensively investigate the sexual differences in external morphological traits, appendicular muscle mass, and internal organ mass of *K. borealis* collected in the type locality during the breeding season. Our findings will provide novel insights into its behavioral, physiological, and reproductive strategies.

## 2. Materials and Methods

### 2.1. Animals

**Figure 1:**
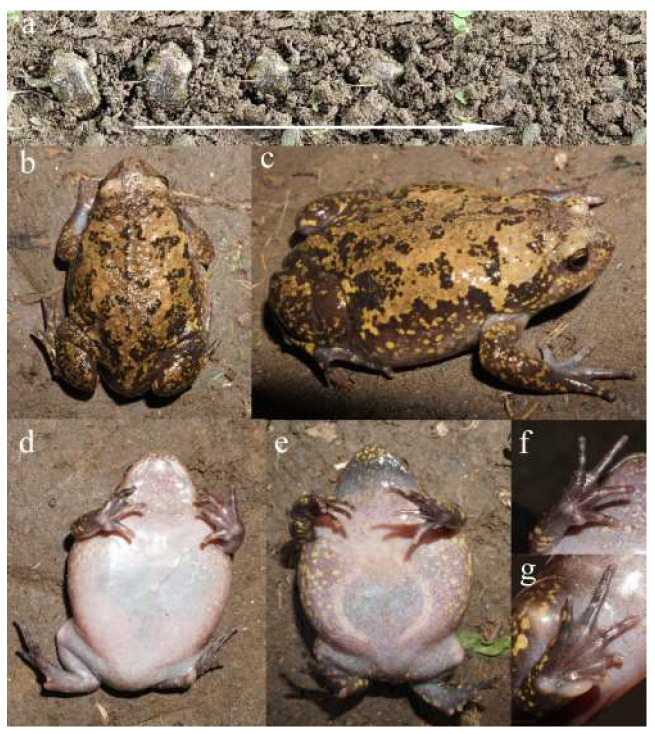
Live photographs documenting the hindlimb-digging processes of *Kaloula borealis* (**a**). The dorsal (**b**), dorsolateral (**c**), and ventral (**d**) views of the female. The ventral view (**e**) of the male. The ventral views of the female hand (**f**) and foot (**g**).

A total of 48 specimens (24 males and 24 females) were randomly collected in July 2024 from Gushan Town, Dandong City, Liaoning Province, China (39°55′39.23″ N, 123°34′08.74″ E, 31.29 m). The females were determined by visible oocytes through the skin of the abdomen, while the males were identified by the external subgular vocal sac, two linea musculina, and thickened ventral glands [41] (Figure 1). Each individual was weighed using an electronic balance (GasonGS-100, Shenzhen, China) to the nearest 0.1 g. They were promptly taken to the laboratory and sacrificed with 2% MS-222 (concentrated tricaine methanesulphonate).

### 2.2. 3D Reconstruction

**Figure 2:**
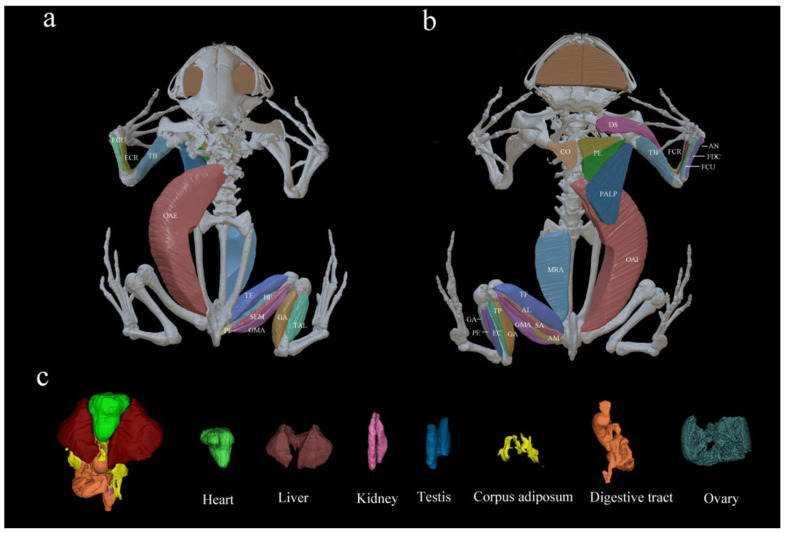
Three-dimensional reconstruction of the studied muscles in the dorsal (**a**) and ventral (**b**) views, as well as internal organs (**c**) of *Kaloula borealis*.

To better visualize and present the studied muscles and internal organs, one male (D20240723011) and one female (D20240723001) were stained in 2% potassium iodide solution for 7–10 days to facilitate tissue contrast enhancement [42,43]. Subsequently, these specimens were wrapped in plastic wrap and scanned in a micro-CT scanner (Micro-CT, PerkinElmer^®^, Waltham, MA, USA); the scanning parameters were set as follows: high-precision scanning mode, voltage of 220 V, current of 60 mA, 14 min. Mimics 21.0 (Materialise HQ Technologielaan, Leuven, Belgium) modeling software was used to extract and transform the original model and complete the 3D reconstruction (Figure 2).

### 2.3. External Morphological Characteristics

In total, 16 functional morphological characteristics were measured using a digital calliper (DEGUQMNT MNT-200, Shanghai, China) to the nearest 0.01 mm. Among them, the snout-vent length (SVL) and body mass (BM) are linked to morphology, physiology, functional ecology, and life history [44]. The head length (HL) and head width (HW) are associated with biological characteristics, such as food preference, and combat behavior [45]. The eye diameter (ED) is related to vision [46]. The upper arm length (UAL), upper arm width (UAW), lower arm and hand length (LAHL), lower arm width (LAD), thigh length (THIL), thigh width (THIW), tibia length (TL), tibia width (TW), tarsal length (TARL), tarsal width (TARW), and foot length (FL) are indicative of locomotor performance [47]. The terminology, abbreviations, and measurements followed those of Fei et al. (2009) and Watters et al. (2016) [41,48].

### 2.4. Appendicular Muscle Mass

For each specimen, we dissected 32 muscles from the right forelimb, pectoral girdle, abdomen, and hindlimb. These muscles were categorized into 12 functional groups based on the literature (Table 1). Each dissected muscle was placed in a labeled centrifuge tube and dried at a constant temperature in a drying thermostat oven (Shanghai Qixin Scientific Instruments Co., Ltd., Shanghai, China) at 60 °C for 48 h until the mass was constant enough to remove moisture. The dried muscle samples were then weighed using an electronic balance with the nearest to 0.1 mg [49].

### 2.5. Internal Organ Mass

Eight internal organs, including the heart (HE), liver (LI), spleen (SP), lung (LU), kidney (KI), corpus adiposum (COAD), testis (TE), and digestive tract (DI), were dissected. These organs were dried at 60 °C at a constant temperature in the same thermostat oven for 48 h until the constant mass was achieved. The dried organ samples were then weighed using an electronic balance to the nearest 0.1 mg. The nomenclature of each organ followed the published literature [60].

### 2.6. Statistical Analyses

First, to meet assumptions of normality and homogeneity of variances, all variables (i.e., external morphological characteristics, muscle mass, and internal organ mass) were log_10_ transformed. Second, to assess sexual dimorphism in functional traits, an independent sample *t*-test was conducted to compare the snout-vent length and body mass between the sexes. Age may influence morphological characteristics, but there is a high correlation between age and body size in anuran species [61,62]. To reduce the age and body size impact, we performed analysis of covariance for the other variables in IBM SPSS Statistics 28 [63], with SVL as a covariate, sex as the fixed factor, and the remaining variates as dependent variables. Third, to examine sex-based morphological differentiation, we performed principal component analysis (PCA) using the “stats” package version 4.4.1 [64], and visualized results using the “ggplot2” package version 3.5.1 in R software version 4.3.2 [65]. Finally, to examine how SVL influences functional trait variation in *K. borealis*, we conducted allometric analyses via linear regression using Origin 2024, modeling significant sexual traits against SVL.

## 3. Results

### 3.1. Results of Statistical Analysis

#### 3.1.1. External Morphological Comparisons (Table 2)

Females exhibited significantly greater SVL (1.63 ± 0.04 mm vs. 1.61 ± 0.02 mm; *p* < 0.01) and eye diameter (0.53 ± 0.04 mm vs. 0.49 ± 0.05; *p* < 0.05) than males. In contrast, males showed greater head width (1.10 ± 0.03 mm vs. 1.09 ± 0.03 mm; *p* < 0.05) and thigh width (0.59 ± 0.04 mm vs. 0.58 ± 0.05 mm; *p* < 0.05) than females. No significant differences were detected in the remaining 12 traits.

**Table 2:** Sexual differences in external morphological traits of *Kaloula borealis*.

External Morphological Characteristics	Mean ± Standard Deviation		R^2^	F	*p*
	Males	Females			
Independent sample *t*-test					
1. Snout-vent length	1.61 ± 0.02	1.63 ± 0.04	/	9.266	**0.03**
2. Body mass	0.80 ± 0.06	0.83 ± 0.11	/	7.039	0.12
Covariance analysis					
3. Head length	0.95 ± 0.03	0.96 ± 0.04	<0.001	0.189	0.67
4. Head width	1.10 ± 0.03	1.09 ± 0.03	0.003	4.697	**0.04**
5. Eye diameter	0.49 ± 0.05	0.53 ± 0.04	0.009	4.091	**0.05**
6. Upper arm length	0.96 ± 0.03	0.97 ± 0.03	<0.001	0.321	0.57
7. Upper arm width	0.42 ± 0.06	0.41 ± 0.06	0.004	1.434	0.24
8. Lower arm width	0.33 ± 0.05	0.32 ± 0.05	0.005	2.400	0.13
9. Lower arm and hand length	1.27 ± 0.02	1.37 ± 0.39	0.174	2.211	0.14
10. Thigh width	0.59 ± 0.04	0.58 ± 0.05	0.010	6.470	**0.01**
11. Tibia length	1.08 ± 0.03	1.09 ± 0.03	<0.001	0.731	0.40
12. Tibia width	0.49 ± 0.05	0.50 ± 0.06	<0.001	0.033	0.86
13. Tarsal length	0.86 ± 0.03	0.87 ± 0.04	<0.001	0.009	0.93
14. Tarsal width	0.42 ± 0.03	0.44 ± 0.02	<0.001	0.355	0.56
15. Foot length	1.21 ± 0.02	1.20 ± 0.03	0.001	3.865	0.06
16. Thigh length	1.18 ± 0.03	1.19 ± 0.03	<0.001	0.249	0.62

Note: All in mm, except body mass in g. Levels of significance *p* less than 0.05 reported in bold. / indicates that the independent sample *t*-test analysis did not produce R^2^ data.

#### 3.1.2. Appendicular Muscle Mass Comparisons (Table 3)

Among the forelimb, abdomen, and pectoral girdle muscles, five of sixteen muscles exhibited sexually dimorphic dry mass (*p* < 0.05). Specifically, males showed significantly greater mass in four muscles: the musculus rectus abdominis, obliquus abdominis internus, anconaeus, and obliquus abdominis externus. Conversely, females demonstrated significantly higher mass in the flexor digitorum communis.

For the hindlimb muscles, five of sixteen muscles displayed significant sexual dimorphism (*p* < 0.05). Specifically, biceps femoris, gracilis major, semimembranosus, semitendinosus, and tibialis posticus were significantly greater in males than in females.

**Table 3:** Covariance analysis of sexual differences in the dry appendicular muscle mass of *Kaloula borealis*.

Appendicular Muscles	Mean ± Standard Deviation of Dry Mass (mg)		R^2^	F	*p*
	Males	Females			
Pectoral girdle muscles					
1. Coracobrachialis longus	0.03 ± 0.21	0.06 ± 0.13	0.001	0.025	0.88
2. Coracobrachialis brevis	0.20 ± 0.17	0.19 ± 0.13	0.009	0.441	0.51
3. Deltoideus scapularis	0.51 ± 0.09	0.52 ± 0.12	0.016	3.316	0.08
4. Pectoralis	0.37 ± 0.12	0.40 ± 0.14	0.010	0.089	0.77
5. Pectoralis abdominis, lateral portion	0.39 ± 0.09	0.42 ± 0.11	0.002	0.384	0.54
6. Coracoradialis	0.27 ± 0.09	0.26 ± 0.11	0.022	2.588	0.12
Forelimb muscles					
1. Flexor carpi radialis	0.02 ± 0.22	−0.03 ± 0.29	0.069	1.065	0.31
2. Flexor digitorum communis	0.03 ± 0.13	0.14 ± 0.11	0.087	6.122	**0.02**
3. Anconaeus	0.19 ± 0.15	0.06 ± 0.16	0.314	16.137	**0.01**
4. Extensor carpi radialis	0.17 ± 0.17	0.11 ± 0.11	0.062	3.280	0.08
5. Extensor carpi ulnaris	0.03 ± 0.15	0.12 ± 0.16	0.014	0.782	0.38
6. Triceps brachii	0.76 ± 0.09	0.81 ± 0.12	<0.001	0.005	0.95
7. Flexor carpi ulnaris	0.00 ± 0.12	0.07 ± 0.16	0.007	0.401	0.53
Abdominal muscles					
1. Musculus rectus abdominis	0.82 ± 0.13	0.69 ± 0.10	0.260	29.458	**0.01**
2. Obliquus abdominis externus	1.59 ± 0.11	0.90 ± 0.16	5.170	676.250	**0.01**
3. Obliquus abdominis internus	1.62 ± 0.24	1.02 ± 0.13	4.327	145.663	**0.01**
Hindlimb muscles					
1. Biceps femoris	0.27 ± 0.12	0.21 ± 0.14	0.103	7.305	**0.01**
2. Triceps femoris	1.33 ± 0.07	1.36 ± 0.11	0.002	0.661	0.42
3. Adductor longus	0.46 ± 0.09	0.52 ± 0.15	0.005	0.396	0.53
4. Sartorius	0.26 ± 0.14	0.27 ± 0.13	0.005	0.308	0.58
5. Adductor magnus	0.98 ± 0.08	0.99 ± 0.13	0.011	2.288	0.14
6. Gracilis major	0.84 ± 0.07	0.83 ± 0.09	0.029	8.396	**0.01**
7. Gracilis minor	0.46 ± 0.11	0.44 ± 0.19	0.048	2.5475	0.12
8. Semimembranosus	0.59 ± 0.09	0.56 ± 0.11	0.053	8.796	**0.01**
9. Semitendinosus	0.64 ± 0.07	0.63 ± 0.11	0.021	4.943	**0.03**
10. Piriformis	0.07 ± 0.11	0.09 ± 0.19	<0.001	0.007	0.93
11. Iliopsoas	0.30 ± 0.12	0.27 ± 0.19	0.075	4.008	0.05
12. Tibialis anticus longus	0.79 ± 0.08	0.81 ± 0.12	0.007	1.384	0.25
13. Tastrocnemius	0.87 ± 0.14	0.90 ± 0.09	<0.001	0.022	0.88
14. Peroneus	0.54 ± 0.09	0.58 ± 0.10	<0.001	0.005	0.95
15. Extensor cruris	0.03 ± 0.09	0.06 ± 0.11	<0.001	0.008	0.93
16. Tibialis posticus	0.05 ± 0.09	0.01 ± 0.16	0.079	7.399	**0.01**

Note: Levels of significance *p* less than 0.05 reported in bold.

#### 3.1.3. Internal Organ Mass Comparisons (Table 4)

Regarding the dry mass of the internal organs, the heart and lung of males were significantly greater than those of females (*p* < 0.05).

**Table 4:** Covariance analysis of sexual differences in the dry internal organ mass of *Kaloula borealis*.

	Mean ± Standard Deviation of Dry Mass (mg)		R^2^	F	*p*
	Males	Females			
1. Heart	1.25 ± 0.15	1.22 ± 0.21	0.112	6.778	**0.01**
2. Liver	1.58 ± 0.15	1.64 ± 0.29	0.015	0.534	0.47
3. Spleen	−0.61 ± 0.35	−0.77 ± 0.29	0.386	3.934	0.06
4. Lung	1.11 ± 0.09	1.04 ± 0.11	0.138	24.952	**0.01**
5. Kidney	0.89 ± 0.06	0.94 ± 0.14	0.002	0.169	0.68
6. Digestive tract	1.67 ± 0.10	1.71 ± 0.14	0.001	0.072	0.80
7. Corpus adiposum	0.37 ± 0.65	0.24 ± 0.90	0.650	1.140	0.29
8. Vesica fellea	−0.39 ± 0.51	−0.49 ± 0.34	0.154	0.780	0.38

Note: Levels of significance *p* less than 0.05 reported in bold.

### 3.2. Results of Principal Component Analysis (Table 5; Figure 3)

**Figure 3:**
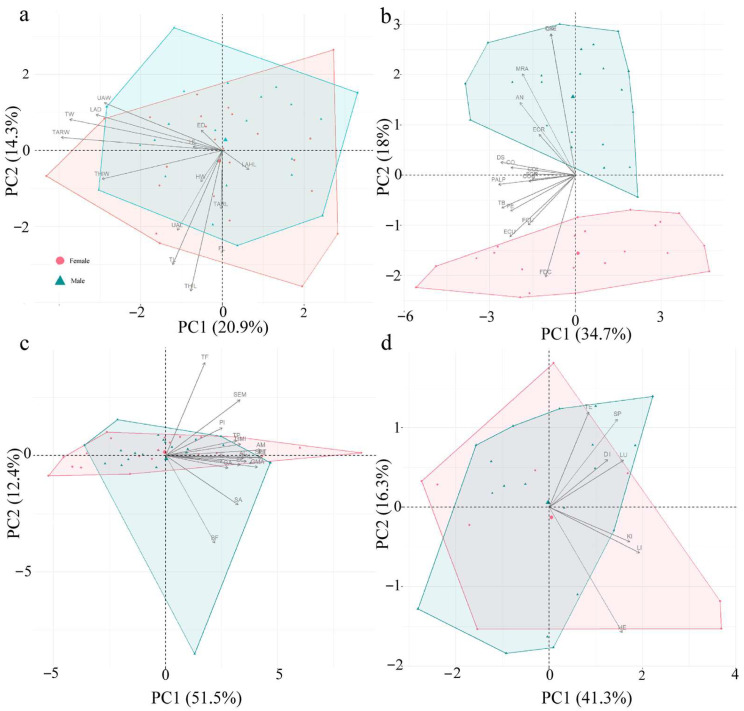
Principal component analysis (PCA) on the external morphological characteristics (**a**), forelimb, abdomen, and pectoral girdle muscle mass (**b**), hindlimb muscle mass (**c**), and internal organ mass (**d**) of males (green triangles) and females (pink circles) of fossorial *Kaloula borealis*.

The PCA results of the external morphological characteristics reveal that the first six principal components collectively explained 72.30% of the total variance. PC1 and PC2 were the primary sources of variation, though neither showed clear sexual distinction. PC1 explained 20.87% of the variance and was primarily associated with size-related variation, with the highest loadings from tibia width (TW, 0.82) and tarsal width (TARW, 0.86). PC2 accounted for 14.33% of the variance and was mainly related to locomotor-related traits, with the highest loading from thigh length (THIL, 0.81). PC3–PC6 revealed independent differentiation in foot and head structures, but their contributions were relatively low, likely due to the overall morphological similarity between male and female individuals (Figure 3a).

The PCA results of the forelimb, abdomen, and pectoral girdle muscles show that the first four principal components collectively explained 67.88% of the total variance. PC1 (34.64%) highlighted strong associations with mating-related adaptations, showing the highest loadings on the pectoralis abdominis lateral portion (PALP, 0.82) and deltoideus scapularis (DS, 0.81). PC2 (18.09%) was primarily associated with respiratory function dominated by abdominal muscles (OAE, 0.85; OAI, 0.85). Notably, obvious sexual dimorphism was observed along both the PC1 and PC2 axes. PC3 and PC4 explained only 16.10% of the total variation combined (Figure 3b). Regarding the hindlimb muscles, the first three principal components explained 71.07% of the variance. PC1 (51.51%) reflected overall hindlimb strength and stability, with the highest loadings on the tibialis anticus longus (TAL, 0.93), adductor magnus (AM, 0.91), and semitendinosus (SET, 0.91). PC2 (12.45%) revealed distinct locomotor patterns through opposing loadings on the tibialis posticus (TF, 0.86) and biceps femoris (BF, −0.81), with these muscles primarily driving variation along this axis. PC3 (7.11%) was weakly associated with iliopsoas (IL, 0.68), potentially reflecting hip joint mobility (Figure 3c).

The PCA results of the internal organs, the first three principal components collectively explain 72.79% of the variance. PC1 (41.29%) reflected the metabolic functions of organs, with the highest loadings from the liver (LI, 0.82) and kidney (KI, 0.91). PC2 (16.28%) was correlated with circulatory function, with highest loading from the heart (HE, −0.67). There was no obvious sexual distinction between the PC1 and PC2 axes. PC3 (15.23%) was associated with digestive functions, with the highest loading from the digestive tract (DI, 0.72) (Figure 3d).

### 3.3. Results of Allometric Analysis for Significantly Sexual Different Traits (Figure 4)

**Figure 4:**
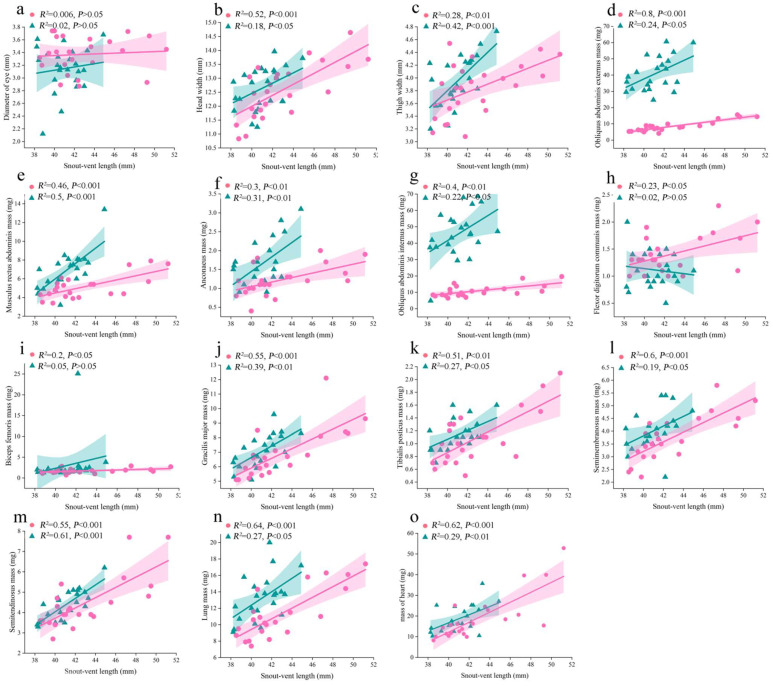
Scatter plots with linear regression lines illustrating the correlation between snout-vent length (X axis) and the external morphological characteristics, limb muscle mass, and internal organ mass (Y axis), which exhibit significant sexual dimorphism in fossorial *Kaloula borealis*. The external morphological characteristics include the eye diameter (**a**), head width (**b**), and thigh width (**c**). The limb muscle mass include the obliquus abdominis externus (**d**), musculus rectus abdominis (**e**), anconaeus (**f**), obliquus abdominis internus (**g**), flexor digitorum communis (**h**), biceps femoris (**i**), gracilis major (**j**), tibialis posticus (**k**), semimembranosus (**l**), and semitendinosus (**m**). The internal organ mass includes the lung (**n**) and heart (**o**). The green triangles indicate the male data, while the pink circles indicate the female data.

For the external morphological characteristics, both the head width and thigh width were significantly correlated with the snout-vent length (*p* < 0.05) (Figure 4a,b). However, there was no significant correlation between the eye diameter and snout-vent length (*p* > 0.05) (Figure 4c).

For the five forelimb and abdominal muscles, the allometric results show that only the flexor digitorum communis in males showed no significant correlation with snout-vent length (*p* > 0.05; Figure 4d–h). For the five hindlimb muscles, the allometric results show that only the mass of biceps femoris in males showed no significant correlation with snout-vent length (*p* > 0.05) (Figure 4i–m).

For the internal organs, the mass of lung and heart were strongly and significantly correlated with snout-vent length in both females and males (*p* < 0.05) (Figure 4n–o).

## 4. Discussion

### 4.1. Sexual Dimorphism in the External Morphological Characteristics

Body size is a fundamental trait that influences virtually all aspects of an organism’s physiology, life history, resource use, and ecological strategy [66]. The degree of body size dimorphism can be modulated by multiple biological factors, including fecundity, reproductive strategies, and growth trajectories [67]. *K. borealis* exhibits female-biased size dimorphism, which may increase the abdominal cavity volume, accommodate more eggs, and boost reproductive output [41].

Vertebrate head size is associated with various biological functions, such as bite force [68], calling behavior [66], and combat [69]. The sexual dimorphism in head morphology could reflect divergent feeding strategies and niche partitioning [70]. Compared to the females, male *K. borealis* possess a significantly broader head, which may improve prey manipulation efficiency and facilitate ingestion of larger arthropods to meet the energetical demands of male reproductive behaviors (e.g., prolonged calling) [71,72]. In addition, the food niche differentiation effectively reduces intraspecific competition [73]. Huang et al. (2019) [74] reported that body size is the strongest predictor of eye size, with larger animals typically having absolutely larger eyes compared to smaller animals. In this study, the larger eye diameter observed in females is consistent with their report.

Robust hindlimbs can enhance power output during locomotion [75]. Therefore, we hypothesize that the significantly wider thigh in male *K. borealis* may generate forceful kicking maneuvers during intrasexual contests, facilitating mating. These functional advantages align with the sexual dimorphism in hindlimb muscle mass.

### 4.2. Sexual Dimorphism in the Appendicular Muscle Mass

Skeletal muscle is a critical component of the motor system and plays a vital role in the survival and reproduction of anurans [76]. Reproductive activities exert significant selective pressure on the appendicular muscle morphology, driving adaptive divergence between sexes [77]. Among the studied 13 forelimb and pectoral girdle muscle mass, the anconaeus and flexor digitorum communis show significant sexual differences. Specifically, the flexor digitorum communis acts as the carpus flexor and provides substantial force to support body weight. In *K. borealis*, the females, with significantly heavier flexor digitorum communis, may adapt to carry males during amplexus, enhancing female endurance throughout the prolonged carrying process and ensuring successful fertilization [78]. The heavier anconaeus stores more kinetic energy, which can improve shock absorption upon landing, and enhances mechanical support for body stabilization [79]. We therefore hypothesize that the males possess more developed elbow muscles, which could enable them to withstand competitive impacts from rivals and to grasp females more tightly during amplexus [80].

The abdominal muscles functionally support the abdominal cavity and participate in respiratory and calling behaviors [52]. In *K. borealis*, significant sexual dimorphism occurs in the mass of musculus rectus abdominis, obliquus abdominis externus, and obliquus abdominis internus. We propose that males have more developed muscles, enabling them to exhibit strong contractility and adapt to the intense calling behavior required during prolonged breeding periods.

Among the 16 hindlimb muscles examined, five exhibit significant sexual dimorphism. Specifically, males possess significantly heavier gracilis major, biceps femoris, semimembranosus, and semitendinosus. These muscles are functionally associated with knee flexion, which likely facilitate males to make powerful kicking movements, to prevent being taken over during male–male combat [49]. Furthermore, the tibialis posticus aids male foot extension and can lift the inner metatarsal tubercle [60], which is relevant to hindlimb digging [81]. The study of the muscular anatomy of *K. borealis* also enhances our understanding of the relationship between the form and function of vertebrates.

Emerging evidence indicates that sexual dimorphism in vertebrate musculature may arise through a testosterone-mediated multilevel regulatory network. Testosterone directly promotes muscle development by inducing muscle cell hypertrophy, particularly in male vertebrates during breeding seasons [82]. Studies on neotropical poison frogs demonstrate that males with elevated testosterone levels exhibit enhanced muscle mass, competitive capacity, and courtship vocalization intensity [83]. Notably, these phenotypic divergences are sustained not only by hormonal regulation but also through sex-specific gene expression. For example, differentially expressed genes and metabolites in the flexor carpi radialis of *Bufo gargarizans* form an epigenetic regulatory network [77], which reprograms substrate metabolism pathways. In the near future, we will investigate the underlying physiological, biochemical, and molecular mechanisms driving these sex-specific muscle adaptations in *K. borealis*.

### 4.3. Sexual Differences in the Internal Organs

Significant sexual dimorphism is observed in the mass of the heart and lung, with both organs larger in males. The heart exhibits distinct morphological and functional adaptations, including ecological demands, reproductive strategies, and energy expenditure during mating behaviors. Additional factors, such as sex, body temperature, and sexual maturity also exert considerable influence on cardiac size [84]. Compared to females, male *K. borealis* possess substantially heavier hearts, a phenomenon hypothesized to correlate with the demands of high-intensity reproductive behaviors (e.g., vocalization). During the breeding season, the courtship behaviors of males require sustained energy expenditure and elevated blood oxygen levels, reinforcing the necessity for cardiac enlargement [85].

The observed sexual dimorphism in lung mass similarly reflects divergent reproductive strategies. The lung, as the primary respiratory organ, correlates closely with individual activity levels [86]. Male *K. borealis* have significantly greater lung mass than females, corresponding to their heightened respiratory needs during the breeding season [85]. It is hypothesized that at the time of sampling, the females had already completed their energetic investment in oogenesis, whereas the males had entered a phase of intense reproductive activities, requiring increased respiratory efficiency to sustain behaviors, such as vocalization. Elevated oxygen demands from these activities may drive further lung development in males [87]. This dimorphism reflects a fundamental energy allocation trade-off between the sexes. Males invest in enhancing their cardiopulmonary capacity to support the high energy demands of courtship behaviors (e.g., calling, competition), while females prioritize energy allocation to gamete production and ovarian development to maximize direct reproductive output [88].

In addition, age [35], growth rate [89], and fluctuating asymmetry [90] have been reported to influence sexual dimorphism; for instance, females tend to choose males with higher symmetry. Furthermore, pathogens may affect morphological deviations in size and weight indicators of traits, and infection may lead to differences in survival and reproductive success rates between sexes by intensifying physiological stress, expanding immune responses, etc. [91]. Future research should further explore the influence of these factors on anuran sexual dimorphism.

## 5. Conclusions

This study provides the first comprehensive analyses of sexual dimorphism in a fossorial anuran species, *K. borealis*. The results confirm the previous prediction that this species exhibits significant sexual dimorphism in its external morphological features (i.e., SVL, eye diameter, head width, thigh width), appendicular muscle mass (i.e., flexor digitorum communis, anconaeus, musculus rectus abdominis, obliquus abdominis externus, obliquus abdominis internus, biceps femoris, gracilis major, semimembranosus, semitendinosus, tibialis posticus), and internal organ mass (i.e., heart and lung). This phenotypic differentiation is jointly driven by sexual selection, fecundity selection, and niche differentiation, reflecting adaptive changes in male and female individuals in response to reproductive investment and competition. This study contributes to enhancing our understanding of the reproductive strategies, as well as relationship between the form and function of anurans. This study also provides a valuable basis for future investigations into the underlying physiological, biochemical, and molecular mechanisms driving the sex-specific morphological adaptations. Future research needs to further consider the impact of other factors, such as age structure, pathogens, morphological deviations, and asymmetry on sexual dimorphism.

## Figures and Tables

**Table 1:** List of the studied 32 appendicular muscles and their functional implications.

Functional Groups	Muscles	References
1. Pectoral girdle flexors	Deltoideus scapularis (DS); coracobrachialis longus (COL); coracobrachialis brevis (COB)	Duellman and Trueb [50]; Myatt, Crompton, and Thorpe [51].
2. Upper arm flexor	Pectoralis (PE); pectoralis abdominis, lateral portion (PALP);	Zhou [52].
3. Forearm flexor	Coracoradialis (CO)	Abdala and Diogo [53].
4. Carpus flexors	Flexor carpi ulnaris (FCU); flexor carpi radialis (FCR); flexor digitorum communis (FDC)	Manzano, Abdala, and Herrel [54]; Böhmer et al. [55].
5. Elbow extensors	Anconaeus (AN); triceps brachii (TB)	Böhmer et al. [55].
6. Carpus extensors	Extensor carpi ulnaris (ECU); extensor carpi radialis (ECR)	Böhmer et al. [55].
7. Abdominal muscle	Musculus rectus abdominis (MRA); obliquus abdominis internus (OAI), obliquus abdominis externus (OAE)	Zhou [52].
8. Knee flexors	Biceps femoris (BF); semimembranosus (SEM); sartorius (SA); gastrocnemius (GA)	Zhou [52]; Taylor-Burt and Biewener [56].
9. Knee flexors and internal tibial rotators	Gracilis major (GMA); gracilis minor (GMI); semitendinosus (SET)	Chin et al. [57].
10. Hip adductors	Adductor magnus (AM); adductor longus (AL)	Přikryl et al. [58].
11. Knee extensor	Triceps femoris (TF)	Padilla, Courant, and Herrel [59].
12. Long extensors	Peroneus (PE); tibialis anticus longus (TAL); tibialis posticus (TP); extensor cruris (EC)	Padilla, Courant, and Herrel [59].
13. Others	Piriformis (PI); iliopsoas (IL)	Zhou [52].

**Table 5:** Principal component analysis of external morphological characteristics and mass of appendicular muscles and internal organs.

External Morphological Characteristics	PC1	PC2	PC3	PC4	PC5	PC6	Forelimb, Chest, and Abdominal Muscles	PC1	PC2	PC3	PC4	Hindlimb Muscles	PC1	PC2	PC3	Internal Organs	PC1	PC2	PC3
1. HL	0.16	−0.02	0.38	0.61	0.05	−0.34	1. COL	0.46	−0.02	0.69	−0.28	1.BF	0.47	−0.81	0.09	1.HE	0.66	−0.67	−0.01
2. HW	0.12	0.18	−0.43	0.70	−0.22	−0.06	2. COB	0.50	−0.09	−0.52	−0.12	2.TF	0.38	0.86	0.04	2.LI	0.82	−0.24	0.27
3. UAL	0.24	0.46	0.37	−0.27	0.17	−0.52	3. DS	0.81	0.06	0.31	−0.16	3.AL	0.74	−0.03	−0.07	3.SP	0.62	0.48	−0.07
4. UAW	0.63	−0.27	0.15	0.27	0.31	0.25	4. PE	0.67	−0.27	−0.19	−0.20	4.SA	0.69	−0.46	−0.05	4.LU	0.67	0.25	−0.49
5. LAHL	−0.14	0.11	0.61	0.21	0.39	0.29	5. PALP	0.82	−0.13	−0.01	−0.07	5.AM	0.91	0.05	0.03	5.KI	0.73	−0.18	0.18
6. LAD	0.68	−0.21	0.05	−0.05	0.18	−0.22	6. CO	0.69	0.01	−0.26	0.45	6.GMA	0.88	−0.11	0.23	6.DI	0.36	0.51	0.72
7. THIL	0.17	0.81	−0.08	−0.12	0.06	0.29	7. FCR	0.47	−0.08	−0.38	0.20	7.GMI	0.72	0.11	−0.01	7.COAD	0.53	0.25	−0.45
8. THIW	0.65	0.17	−0.45	0.04	−0.19	0.07	8. FCU	0.49	−0.36	−0.27	−0.36	8.SEM	0.71	0.51	0.26				
9. TL	0.27	0.66	0.17	−0.31	−0.17	0.14	9. FDC	0.27	−0.65	0.20	0.18	9.SET	0.91	−0.01	−0.02				
10. TW	0.82	−0.18	0.00	−0.11	−0.16	0.00	10. AN	0.64	0.39	−0.20	0.21	10.PI	0.54	0.26	−0.53				
11. TARL	0.01	0.34	−0.41	0.22	0.65	0.21	11. ECR	0.43	0.20	0.49	0.55	11.IL	0.54	−0.05	0.68				
12. TARW	0.86	−0.08	0.09	−0.09	0.05	0.02	12. ECU	0.68	−0.47	0.01	0.19	12.TAL	0.93	−0.02	−0.06				
13. FL	0.00	0.59	0.21	0.44	−0.28	−0.19	13. MRA	0.62	0.57	0.08	−0.24	13.GA	0.59	−0.12	−0.09				
14. ED	0.11	−0.12	0.45	0.15	−0.44	0.54	14. OAE	0.34	0.85	−0.09	0.07	14.PE	0.77	−0.05	−0.16				
							15. OAI	0.34	0.85	0.02	−0.16	15.EC	0.72	−0.09	−0.45				
							16. TB	0.78	−0.28	0.14	−0.11	16.TP	0.68	0.14	0.15				
Explanatory rate	20.87	14.33	10.88	10.36	8.26	7.59		34.63	18.09	9.51	6.59		51.51	12.45	7.11		41.29	16.28	15.23

## Data Availability

The raw data used in this study can be made available upon request from the corresponding authors.
